# Efficacy of photodynamic therapy using hematoporphyrin derivative nanomedicine on hepatocellular carcinoma cells

**DOI:** 10.7150/jca.97637

**Published:** 2024-09-03

**Authors:** Yuanyuan Cheng, Shushu Gong, Qianqian Li, Juan Shen, Hongjuan Huang

**Affiliations:** 1 Department of Radiology, The Haimen Hospital Affiliated of Nantong University, Nantong 226100, Jiangsu Province, China.; 2 Department of Oncology, The Haimen Hospital Affiliated of Nantong University, Nantong 226100, Jiangsu Province, China.; 3 Department of Pharmacy, The Haimen Hospital Affiliated of Nantong University, Nantong 226100, Jiangsu Province, China.

**Keywords:** disodium gadolinium sulfate, MRI, hematoporphyrin derivatives, nanodrugs, photodynamic therapy, liver cancer

## Abstract

**Objective:** To demonstrate the efficacy of photodynamic therapy (PDT) using hematoporphyrin derivative (HPD) nanomedicine in combination with conventional chemotherapy based on gadolinium-diethylenetriamine pentaacetic acid (Gd-DTPA) magnetic resonance imaging (MRI) for hepatocellular carcinoma (HCC) therapy.

**Methods:** HPD nanomedicine was prepared, and the cytotoxicity of HPD nanomedicine at different concentrations on HCC cells and the half-maximal inhibitory concentration (IC_50_) were analyzed. Sixty HCC patients who visited our hospital from 2021 to 2023 were retrospectively analyzed. Patient data were analyzed, with 30 cases in control group (CG) receiving conventional chemotherapy for HCC, and 30 cases in observation group (OG) receiving conventional chemotherapy combined with HPD nanomedicine PDT. Gd-DTPA MRI was utilized to monitor the morphological and biological characteristics of the lesions in patients. After treatment completion, the long-term efficacy of patients and the levels of bcl-2 and bax proteins in primary HCC cells were evaluated.

**Results:** The IC_50_ values of HPD on HepG2 cell proliferation and the cell inhibition rates gradually increased with increasing doses of HPD (50 μM, 25 μM, 12.5 μM, 6.25 μM, 3.13 μM, 1.56 μM, 0.78 μM). HPD exhibited great anti-proliferative effects on HepG2 cells at relatively low concentrations. The differences in expression rates of bcl-2 protein and bax protein between groups were considerable (*P*<0.05). There were neglectable changes in AST and ALT levels between the two groups before treatment, but they were markedly reduced after treatment versus before treatment (*P*<0.05), with OG showing considerably lower levels than CG after treatment (*P*<0.05). Additionally, patients in OG exhibited better survival rates after the course of treatment versus those in CG.

**Conclusion:** This study demonstrates that the combination of conventional chemotherapy based on Gd-DTPA MRI with HPD nanomedicine PDT greatly improves the efficacy of treatment for HCC patients. This combined treatment strategy not only enhances therapeutic outcomes but also alleviates adverse reactions associated with conventional treatment, providing a novel approach for future research in the treatment of HCC.

## Introduction

Hepatocellular carcinoma (HCC), as a highly malignant tumor, lacks specific symptoms in its early stages, often progressing to intermediate or advanced stages by the time symptoms manifest. Clinical manifestations include fatigue, weight loss, and occasionally fever and jaundice 1. Due to its insidious nature and rapid progression, HCC has long been a major challenge in global public health 2,3. Despite some progress in the field of HCC treatment, its efficacy remains limited, particularly in advanced-stage patients. Traditional approaches such as conventional chemotherapy are hindered by their toxic side effects and limitations, failing to meet patient expectations for treatment outcomes and quality of life. Conventional chemotherapy for HCC yields limited efficacy and is often accompanied by a range of adverse reactions 4,5.

In recent years, photodynamic therapy (PDT) has garnered widespread attention for its attributes of localized treatment, non-invasiveness, and low toxicity 6. PDT employs photosensitive substances that selectively accumulate in tumor cells and are activated by light of a specific wavelength, thus serving as a novel therapeutic modality for tumor targeting and destruction. PDT has pioneered a unique treatment approach for various malignancies in clinical practice, attracting significant attention from the medical community both domestically and internationally. Gunaydin et al. (2021) 7 noted that PDT primarily relies on the generation of singlet oxygen via excitation of photosensitizers, which can effectively disrupt target tumor cells. Relative to conventional treatment modalities, PDT offers advantages such as non-invasiveness, localized therapy, and reduced toxicity, presenting a new treatment option for hepatocellular carcinoma (HCC) patients 8. However, PDT still faces challenges in HCC treatment, including issues related to photosensitizer selection and localized aggregation, which impact its clinical application. Therefore, enhancing the therapeutic efficacy of PDT and addressing its limitations have become crucial directions for current research endeavors. Currently, the photosensitizers utilized in clinical practice or under investigation predominantly belong to the class of porphyrin compounds, among which hematoporphyrin derivatives (HPD) are widely studied and preferred for clinical PDT applications 9. Nanomedicines can actively target tumor tissues by adjusting their size, shape, and surface functionalization, thereby enhancing therapeutic efficacy, controlled release, photothermal effects, and image-guided therapy 10. HPD nanomedicines typically exhibit excellent fluorescent imaging properties, enabling tumor localization and monitoring. Furthermore, nanomedicines enhance magnetic resonance imaging (MRI) contrast, facilitating the monitoring of drug distribution *in vivo* and improving treatment precision 11.

MRI, as an advanced imaging modality, is widely employed in the diagnosis and treatment of tumors. Gadolinium-diethylenetriamine pentaacetic acid (Gd-DTPA) MRI technology, with its highly precise monitoring of the morphological and biological characteristics of tumor lesions, facilitates timely assessment of treatment efficacy 12. With clinicians increasingly demanding more sophisticated imaging techniques, there is a need to detect HCC nodules with malignant potential before the progression of liver cirrhosis, enabling early treatment and improving survival rates for HCC patients 13. Gd-DTPA is the first clinically applied targeted MRI contrast agent, characterized by uptake in normal functional liver cells, resulting in high detection and diagnostic rates. It offers unique advantages in distinguishing multiple steps of carcinoma evolution in liver cirrhosis nodules 14.

This study aimed to investigate the therapeutic efficacy and survival outcomes of combining traditional chemotherapy with HPD nanomedicine PDT in patients with HCC using Gd-DTPA MRI technology. The morphological and biological characteristics of lesions in patients were monitored using Gd-DTPA MRI. Following completion of treatment, long-term efficacy in patients and levels of bcl-2 and bax proteins in primary HCC cells were assessed. Through clinical evaluation and MRI imaging analysis, the impact of this combined treatment strategy on patients with HCC was explored. This endeavor sought to provide more effective and innovative approaches to HCC treatment, ultimately enhancing patients' treatment experiences and prognoses.

## Materials and Methods

### Instruments and reagents

Reagents and materials included HPD (purity 98%, Shanghai Aladdin Bio-Chem Technology Co., Ltd., China); gadoxetic acid (trade name: Primovist, Bayer HealthCare AG, Germany); human HCC cell line SMCC-7721 (Shanghai Kanglong Biotech Co., Ltd., China); dimethyl sulfoxide (DMSO) (Chengdu Kelong Chemical Reagent Factory, China); RPMI-1640 culture powder (Biosharp, China); phosphate-buffered saline (PBS) solution (Solarbio, China).

Instruments included electronic balance (model BP211D, Sartorius AG, Germany); microplate reader (BIO-RAD Corporation); 3-(4,5-dimethylthiazol-2-yl)-2,5-diphenyltetrazolium bromide (MTT) (Sigma-Aldrich Co., Ltd., China); transmission electron microscope (TEM, model Tecnai G2 20, Thermo Fisher Scientific Inc., USA); CO_2_ incubator with 5% CO_2_ and saturated humidity (model Incubator 371, Thermo Fisher Scientific Inc., USA); constant temperature water bath (model SB-1200, Tokyo Rikakikai Co., Ltd., Japan).

### Research ideas

**Figure 1:**
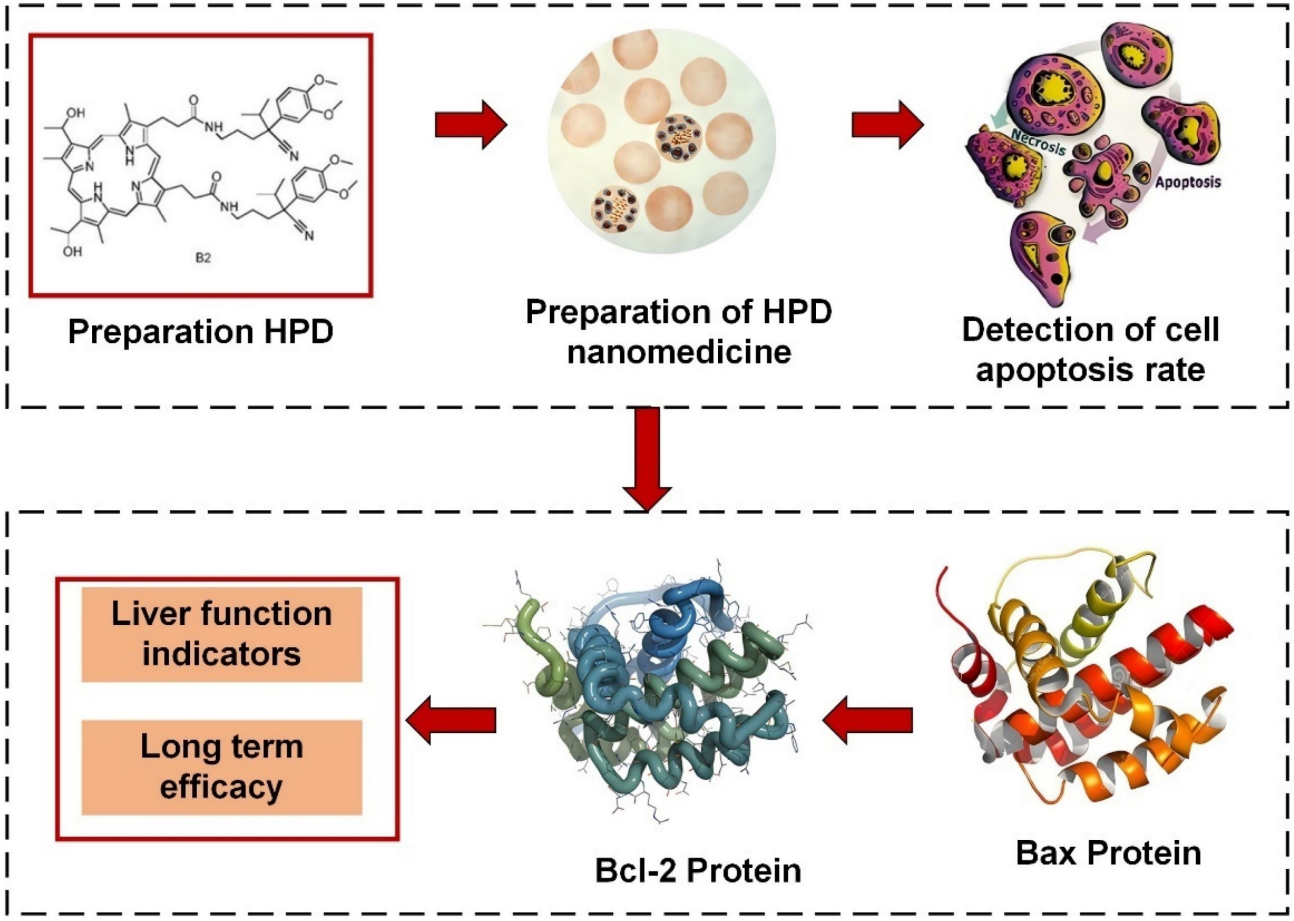
Flowchart of research ideas.

As illustrated in Figure 1, this work is primarily divided into two parts. Firstly, an analysis of HPD nanomedicine was conducted, focusing on parameters such as the cell inhibition rate at different concentrations of HPD nanomedicine and their respective IC_50_ values. Subsequently, the efficacy of conventional chemotherapy combined with HPD nanomedicine PDT based on Gd-DTPA MRI for HCC patients was evaluated. Analytical parameters included the bcl-2 and bax protein levels in primary liver cancer cells, as well as liver function indicators.

### Preparation of HPDs

**Figure 2:**
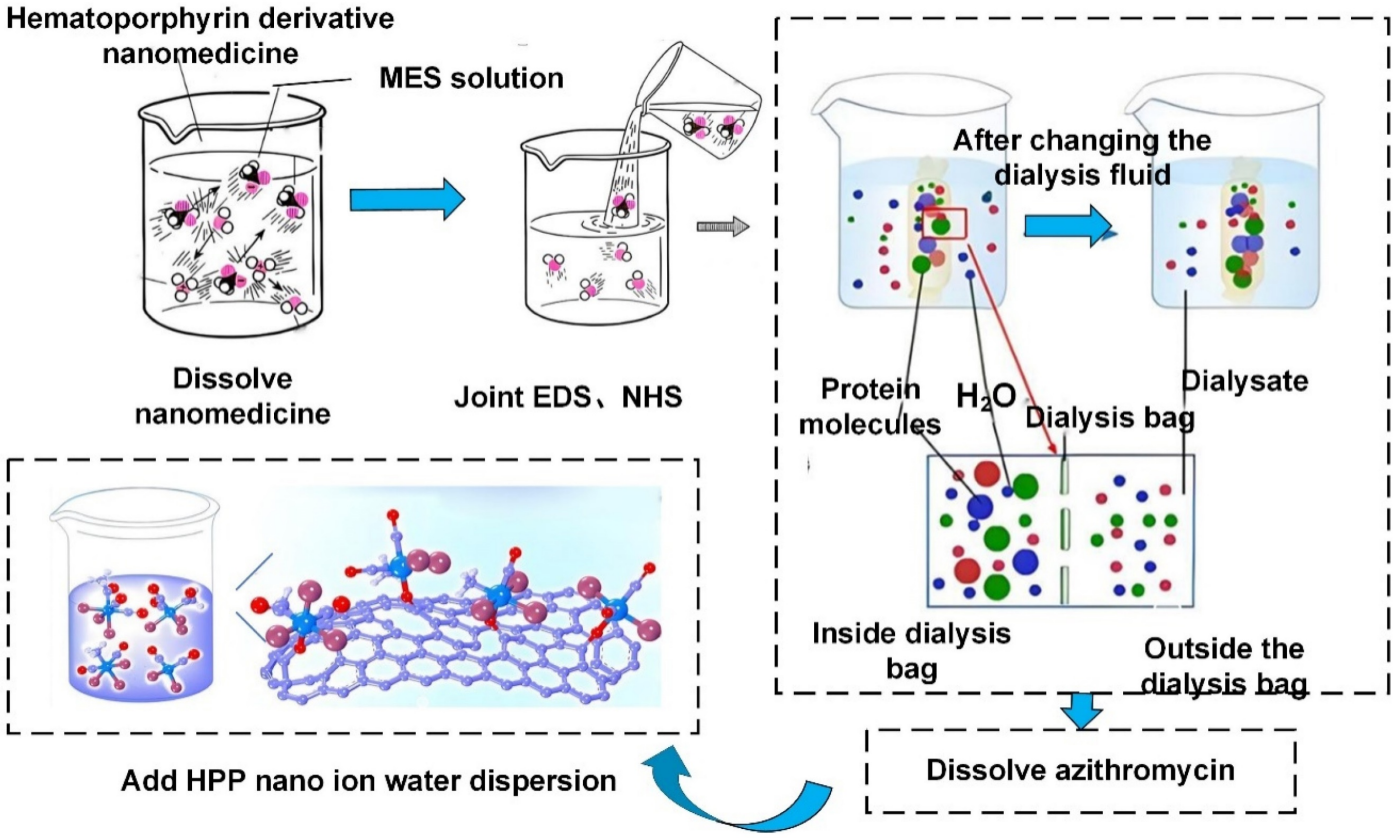
Preparation process diagram of HPD nanomedicine.

In Figure 2, the reaction vessel containing porphyrin (0.5 mmol) was dissolved in dimethylformamide (DMF, 30 mL) and stirred at 25 °C for 3 hours. Upon complete reaction, water (100 mL) was applied to the reaction vessel, and the mixture was placed on ice to allow for the precipitation of the solid. The porphyrin derivatives were separated from impurities via silica gel column chromatography, using a dichloromethane: methanol ratio of 50:1 as the eluent. Eventually, a dark brown solid of pure porphyrin derivatives was obtained.

### Preparation of HPD nanodrugs

Twenty grams of porphyrin derivative nanomedicine were dissolved in a 20 mL solution of 2-(N-morpholino) ethanesulfonic acid (MES) to stabilize the nanomedicine solution at a specific pH. Carboxyl activation groups, EDS and NHS, were applied to the aforementioned solution and stirred, then left at room temperature for 2-8 hours. Subsequently, 40 mg of bis-amine polyethylene glycol was dissolved in 10 mL of PBS solution (pH 7.4). The solution containing carboxyl activation groups EDS and NHS was then applied dropwise to the PBS solution. The materials in the solution were transferred to a dialysis bag with a cutoff molecular weight of 5,000, and dialyzed in borate buffer solution for 48 hours. Twenty milligrams of azithromycin were dissolved in 10 mL of double-distilled water, stirred at 25 °C, and set aside. Then, 2 mL of the prepared azithromycin solution was added dropwise to 5 mL of HPP nanoparticle aqueous dispersion and left overnight at 4 °C. Subsequently, it was placed in a dialysis bag and dialyzed with double-distilled water for 2 hours.

### Cell inhibitory activity experiment

The HCC cell line SMCC-7721 was cultured in a CO_2_-saturated humidified incubator at 37 °C. The adopted medium consisted of RPMI1640 cell culture medium with 10% heat-inactivated fetal bovine serum, 100 U/mL penicillin, and 100 U/mL streptomycin. The medium was replaced after 48 hours, and upon reaching confluency, cells were passaged using 0.25% trypsin. Cells in logarithmic growth phase with good viability were harvested and digested using a digestion solution containing 0.125% trypsin and 0.01% EDTA. After counting, cells were prepared at 2-4×10^4^ cells/mL, and 100 μL of cell suspension was seeded into a 96-well plate.

The plates were then incubated in a constant temperature incubator for 24 hours. Subsequently, the medium was replaced again, this time with fresh one supplemented with varying concentrations of porphyrin derivatives. Each concentration was set up with 5 replicate wells, and cells were cultured for 72 hours. Following this, NTT was applied and cells were incubated for four hours. After removal of the supernatant, DMSO was applied and the plate was shaken on a shaker for 10 minutes. Finally, the absorbance was measured at a wavelength of 570 nm using an ELISA reader, and the cell inhibition rates at each concentration (50 μM, 25 μM, 12.5 μM, 6.25 μM, 3.13 μM, 1.56 μM, 0.78 μM) were calculated. The IC_50_ value was determined using SPSS, with control group (CG) assigned a default value of 100.

Cell Inhibition Rate = (Relative OD value of negative control well - Relative OD value of drug-sensitive well) / OD value of negative CG × 100%

Relative OD value of drug-sensitive well = Absolute OD value of drug-sensitive well - Absolute OD value of blank control well

### Research objects

A retrospective analysis was conducted on 60 cases of HCC patients who visited our hospital from 2021 to 2023 as the study subjects. Observation group (OG) consisted of 30 cases, including 19 males and 11 females, with an average age of (52.28±10.43) years and a disease duration ranging from 1 to 5 years, with a mean duration of (3.17±1.45) years. CG also comprised 30 cases, with 14 males and 16 females, with an average age of (56.81±9.87) years and a disease duration ranging from 1 to 5 years, with a mean duration of (3.34±1.62) years. CG received conventional chemotherapy for liver cancer, while OG received conventional chemotherapy combined with porphyrin derivative nanomedicine PDT. According to the TMN staging criteria 15, there were 25 cases in stage I, 20 cases in stage II, and 15 cases in stage III. There was slight difference in gender and age between groups (*P*<0.05). General data analysis of all patients revealed stable vital signs and no obvious contraindications upon examination. All patients who met the inclusion criteria in this study received trial regulations and signed informed consent forms, which were approved by the ethics committee of The Haimen Hospital Affiliated of Nantong University.

Inclusion criteria: (1) Patients meeting the criteria of the *Primary Liver Cancer - 12th Edition* 16, (2) Inpatients with detailed clinical treatment information collected (including age, gender, past medical history, and chemotherapy history), (3) Patients with complete medical records who have been receiving long-term treatment at The Haimen Hospital Affiliated of Nantong University, (4) Absence of other psychiatric disorders, (5) Absence of extensive cancer cell metastasis, (6) Patients aged >18 years.

Exclusion criteria: (1) Refusal to participate in this study, (2) Incomplete medical records, (3) Presence of contraindications in imaging studies, (4) Presence of observable organ impairments such as in the brain, heart, or kidneys, (5) Patients who withdrew or died during the study, (6) Patients with concomitant malignant diseases.

### MRI detection of gadolinium selenide disodium

All patients underwent radiological examination in the Radiology Department of our hospital, conducted by the same radiologist. For T1-weighted imaging, contrast agent was injected into the antecubital vein at a rate of 1-2 mL/s, followed by a flush of 20-30 mL of 0.9% saline solution through the catheter. Gadopentetate dimeglumine was then intravenously administered to the patients. This was typically done before or during the baseline MRI scan. Gadopentetate dimeglumine, upon entry into the bloodstream, accumulates in tissues, enhancing the contrast of MRI images. Following the injection of gadopentetate dimeglumine, a repeat MRI scan was performed to obtain enhanced images using three-dimensional T1-weighted gradient echo sequences. Breath-holding was instructed when the contrast agent reached the abdominal aorta segment of the patient, to scan the arterial phase. The portal vein phase was acquired at 50s to 80s post-injection, and equilibrium phase images were obtained at 180s. After 20 minutes post-injection, T1-weighted fat-suppressed images, namely hepatobiliary phase images, were acquired. The obtained MRI images were then interpreted and analyzed to assess the patient's condition.

### Therapeutic methods

CG received standard treatment, where 20-30 mg of cisplatin was dissolved in 20 ml of saline solution and intravenously administered over 5 consecutive days, constituting one treatment cycle. Typically, this cycle was repeated every 3-4 weeks, but could also be intermittently administered for 3-4 cycles based on the patient's condition.

OG received combination therapy with porphyrin derivative nanomedicine in addition to the standard treatment received by CG. Initially, the pre-prepared porphyrin derivative nanomedicine was diluted in 100 mL of 5% glucose solution at 5 mg/kg body weight and adopted intravenously via drip infusion. Infusions were given every 7 days, and after 48 hours of administration, PDT was initiated. During PDT, patients remained supine, received local anesthesia with 1% lidocaine, and underwent liver tumor puncture guided by ultrasound. Light with a wavelength of 630 nm and an end diffuser head measuring 1 cm in length, with a power of 300-350 nW, and an accumulated energy of approximately 220 J per dose, was administered once a day for a total of 3 sessions, with a one-day interval between each session, forming one treatment cycle (7 days). The treatment course typically lasted 4-5 weeks. Patients were advised to avoid sunlight exposure for one month after treatment.

### Detection of apoptosis in liver cancer cells

Cell analysis was conducted using flow cytometry. Liver cancer cell punctures were performed under MRI guidance. Primary liver cancer cells were cultured in DMEM filtered through a 0.22 μm membrane filter and maintained at 37 °C, 5% CO_2_, and saturated humidity conditions. Primary liver cancer cells in logarithmic growth phase were selected, suspended, and seeded into 24-well culture plates for 12 hours, followed by an 8-hour incubation in a cell culture incubator. After cell collection, cells were injected with cold ethanol at 4 °C and gently shaken to prevent cell aggregation. The specimens were then centrifuged, ethanol was discarded, and cells were rinsed twice with PBS, which were resuspended in 0.5 mL of PBS, incubated at 37 °C for 30 minutes, and then placed in an ice bath to terminate the action of RNase A and stained for DNA for at least 30 minutes. Cell detection was performed through nylon mesh filtration, and apoptosis of primary liver cancer cells was detected using Annexin V-PI/PI technology. *CellQuest* was used for result analysis, plotting double-parameter dot plots using FIDC and PI fluorescence for apoptosis rate analysis. Additionally, enzyme-linked immunosorbent assay was employed to detect Bcl-2 protein and Bax protein levels.

### Outcome measures

The apoptotic status of primary liver cancer cells and Bcl-2 and Bax protein levels were compared between the two groups. Additionally, statistical analysis was performed to assess the short-term efficacy and long-term survival rates between the groups.

Changes in liver function [ALT (alanine aminotransferase, normal range 5-39 U/L) and AST (aspartate aminotransferase, normal range 5-39 U/L)] before and after completion of the entire treatment course were compared between the two groups.

The efficacy evaluation is shown in Table 1, with total effective rate=complete response rate + partial response rate + improvement rate.

### Statistics

Employing *SPSS 19.0*, data were denoted as x'±s (where x' represents the mean value of the x data set). The* t*-test was used for analyzing differences between groups, while one-way analysis of variance was employed for comparisons among groups. *P* < 0.05 indicated statistically significant.

## Results and Discussion

### HPD nanomedicine

**Figure 3:**
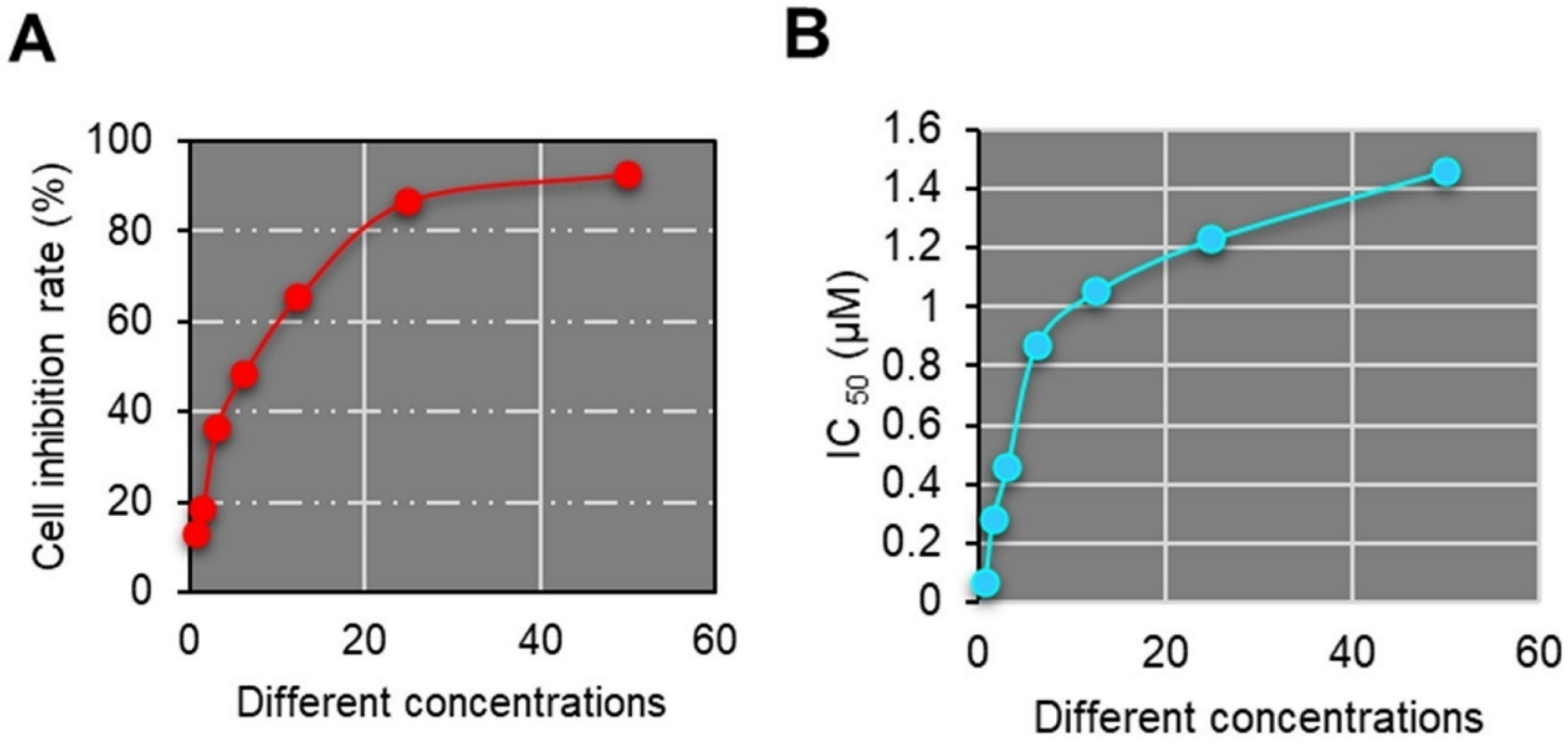
HPD nanomedicine cell inhibition rate and IC_50_ value.

Porphyrins are endogenous porphyrins produced by the acidic hydrolysis of hemoglobin. They are photosensitizers, selectively absorbed and retained in malignant tumors. Studies have demonstrated that HPD nanomedicine exhibits excellent therapeutic efficacy and safety in various tumor models 16. Porphyrin molecules can be absorbed and retained by tumor cells. Due to the tenfold higher affinity of tumor cells for porphyrins compared to normal cells, the concentration of porphyrins in tumor cells is much higher than in normal tissues. Research by Areseney et al. (2007) 17 indicated that porphyrin derivative nanomedicines can significantly reverse multidrug resistance in tumors, restoring cell sensitivity to anticancer drugs. The combined application of anticancer drugs with porphyrin derivative nanomedicines can restore the sensitivity of drug-resistant cells to anticancer drugs, achieving therapeutic and control effects on tumors 18,19. In Figure 3, the IC_50_ of HPD on primary liver cancer cell proliferation at various concentrations (50 μM, 25 μM, 12.5 μM, 6.25 μM, 3.13 μM, 1.56 μM, 0.78 μM) and the gradual enhancement of cell inhibition rate with increasing doses were demonstrated. HPD exhibited marked anti-proliferative effects on primary liver cancer cells even at lower concentrations, indicating its potential anticancer activity, particularly in inhibiting primary liver cancer cell proliferation. The findings of this study indicated that HPD can effectively inhibit tumor cell proliferation even at low doses, thereby reducing potential toxicity risks. This holds significant clinical implications, as the efficacy of low-concentration drugs can minimize damage to normal cells, reduce treatment side effects, and improve patient tolerance. The significant anti-proliferative effect of HPD at low concentrations suggests the potential for achieving effective and low-toxicity treatment outcomes in clinical applications. Further enhancement of the prospects for HPD nanomedicine in cancer treatment can be achieved through improving its pharmacokinetic properties, enhancing tumor targeting, and reducing side effects.

### Primary liver cancer cell apoptosis detection

**Figure 4:**
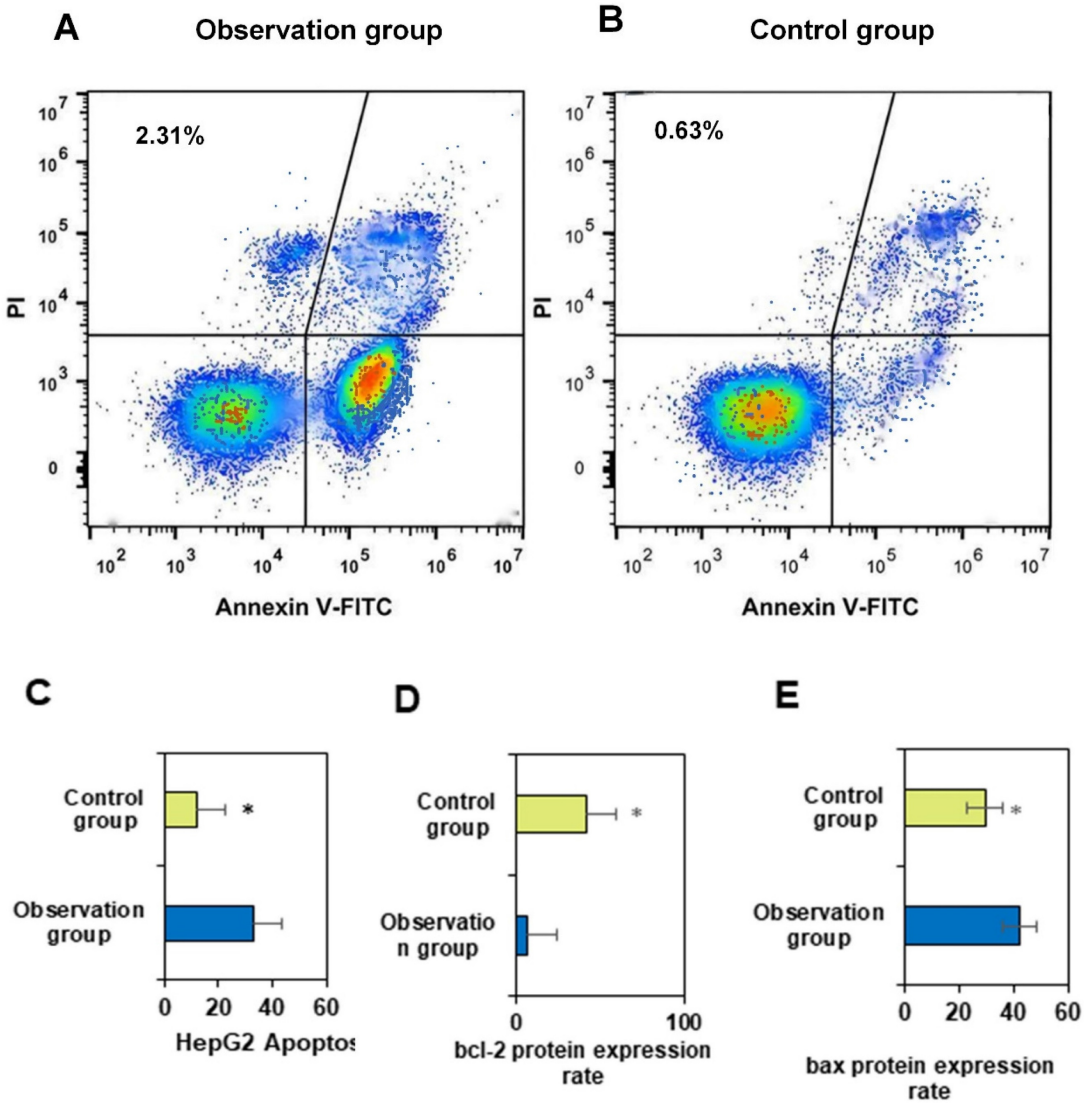
** FITC AnnexinV/PI fluorescence double staining cell apoptosis effect diagram and the expression rate of bcl-2 protein and bax protein.** A and B shows the effect of FITC AnnexinV/PI fluorescence double staining on cell apoptosis; C shows HepG2; D shows the expression rate of bcl-2 protein; E shows the expression rate of bax protein. (*P<0.05 vs. CG)

Annexin V-FITC single-positive cells represent early apoptotic cells, while cells double-positive for Annexin V-FITC and PI staining are indicative of necrotic cells or late-stage apoptosis. Cells positive only for PI staining indicate cells with damaged membranes. Measured data may vary depending on cell type and apoptosis status. Following staining with the FITC-Annexin V/PI fluorescence dual staining apoptosis detection kit, the results are presented in Figure 4A for OG and Figure 4B for CG.

The apoptotic rate of primary liver cancer cells in OG was markedly superior to that in CG (*P*<0.05). PDT with porphyrin derivative nanomedicines may induce cell apoptosis by generating reactive oxygen species through the action of photosensitizers. This treatment modality may have a greater impact on liver cancer cells, leading to an increase in apoptotic rate. Porphyrin derivative nanomedicines may effectively target cancer cells during treatment, enhancing therapeutic efficacy. This may render liver cancer cells more susceptible to treatment, resulting in an elevated apoptotic rate 20. Studies by Yu et al. (2023) 21 and Heer et al. (2022) 22 demonstrated the significant efficacy of photodynamic therapy in bladder treatment. The results of this study indicated that the combination of conventional treatment and PDT with porphyrin derivative nanomedicines may synergistically induce more cell apoptosis at the cellular level. Liver cancer cells may be more sensitive to specific treatment modalities, and the treatment regimen in OG may better align with the biological characteristics of liver cancer cells, thus leading to an increased apoptotic rate 23,24. In summary, the apoptotic rate of primary liver cancer cells in OG was substantially superior to that in CG, suggesting that PDT and drug targeting may play a key role in increasing the apoptotic rate.

The differences in Bcl-2 and Bax proteins between two groups shown in Figures 4D and 4E were considerable (*P*<0.05). This may be attributed to the differential effects of different treatment regimens on the cellular apoptotic pathway, and further experiments and in-depth studies are needed to elucidate the specific molecular mechanisms. Bcl-2 is an anti-apoptotic protein, and its high expression is typically associated with enhanced cellular anti-apoptotic capability. OG received PDT with porphyrin derivative nanomedicines, which may lead to a decrease in the expression level of Bcl-2 protein, as this treatment modality may facilitate the entry of tumor cells into the apoptotic pathway.

Bax is a pro-apoptotic protein, and its high expression is typically associated with enhanced cellular apoptotic capability. OG may have increased the expression level of Bax protein by combining PDT with porphyrin derivative nanomedicines, thereby promoting cell apoptosis. PDH nanomedicine PDT may activate the cellular apoptotic pathway, including the regulation of the Bcl-2 family of proteins, thereby affecting Bcl-2 and Bax expression. This may render liver cancer cells in OG more susceptible to apoptosis. The treatment regimen in OG may have a synergistic effect on the expression of Bcl-2 and Bax, making the changes in these two proteins more notable. This may be the result of the interaction between conventional treatment and PDT. The treatment regimen in OG may better align with the biological characteristics of liver cancer cells, leading to differences in the expression of Bcl-2 and Bax.

### Comparison of long-term therapeutic effects between two groups of patients

**Figure 5:**
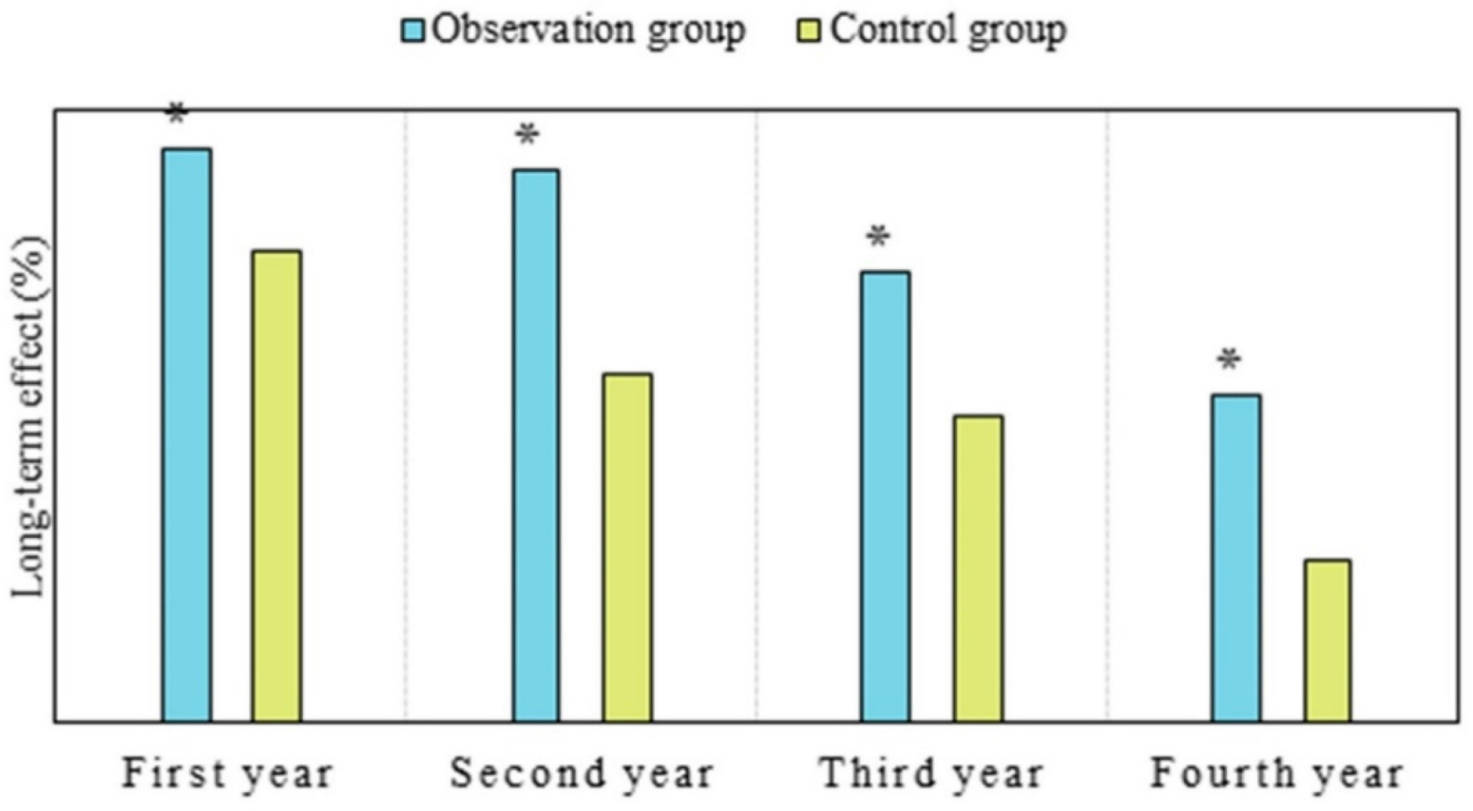
** Comparison of long-term efficacy between two groups of patients.** (Note: *P<0.05 vs. CG)

PDH is a first-generation photosensitizer that can remain in the body for a relatively long time. Currently, in clinical practice, PDH is believed to produce singlet oxygen after light exposure, which is crucial for killing tumor cells. Its main mechanism of action is related to oxidative damage to cellular membranes, lipids, and nuclear functions 25,26. As shown in Figure 5, the long-term efficacy in OG in the first year was 93.33%, versus 76.67% in CG; in the second year, it was 90.0% in OG versus 56.67% in CG; in the third year, it was 73.33% in OG versus 50.0% in CG; and in the fourth year, it was 53.33% in OG versus 26.67% in CG. The efficacy of OG was notably superior to that of CG in the first, second, third, and fourth years (*P* < 0.05). Considering the reasons, nanoparticles enable more drug delivery into tumor tissues, even penetrating tumor cells to exert anticancer effects. Additionally, nanoparticles possess characteristics of sustained release, prolonging the retention time of drugs in tumors, which is consistent with findings from Moffatt and Cristiano (2006) 27 and Liang et al. (2008) 28. This study demonstrated significant therapeutic efficacy of PDH in tumor patients. This highlights the advantages of nanomedicine in tumor treatment, as many studies have indicated that nanoparticles are more likely to penetrate tumor cell gaps, extend circulation time in the body, prolong drug action duration, and reduce drug toxicity. Currently, research on PDH nanomedicine therapy is relatively limited, and knowledge regarding tissue photodynamic damage mechanisms is still lacking. Future studies should explore the therapeutic efficacy and safety of PDH nanomedicine in liver cancer based on different photosensitizers as needed.

### Comparison of liver function indicators between two groups

**Figure 6:**
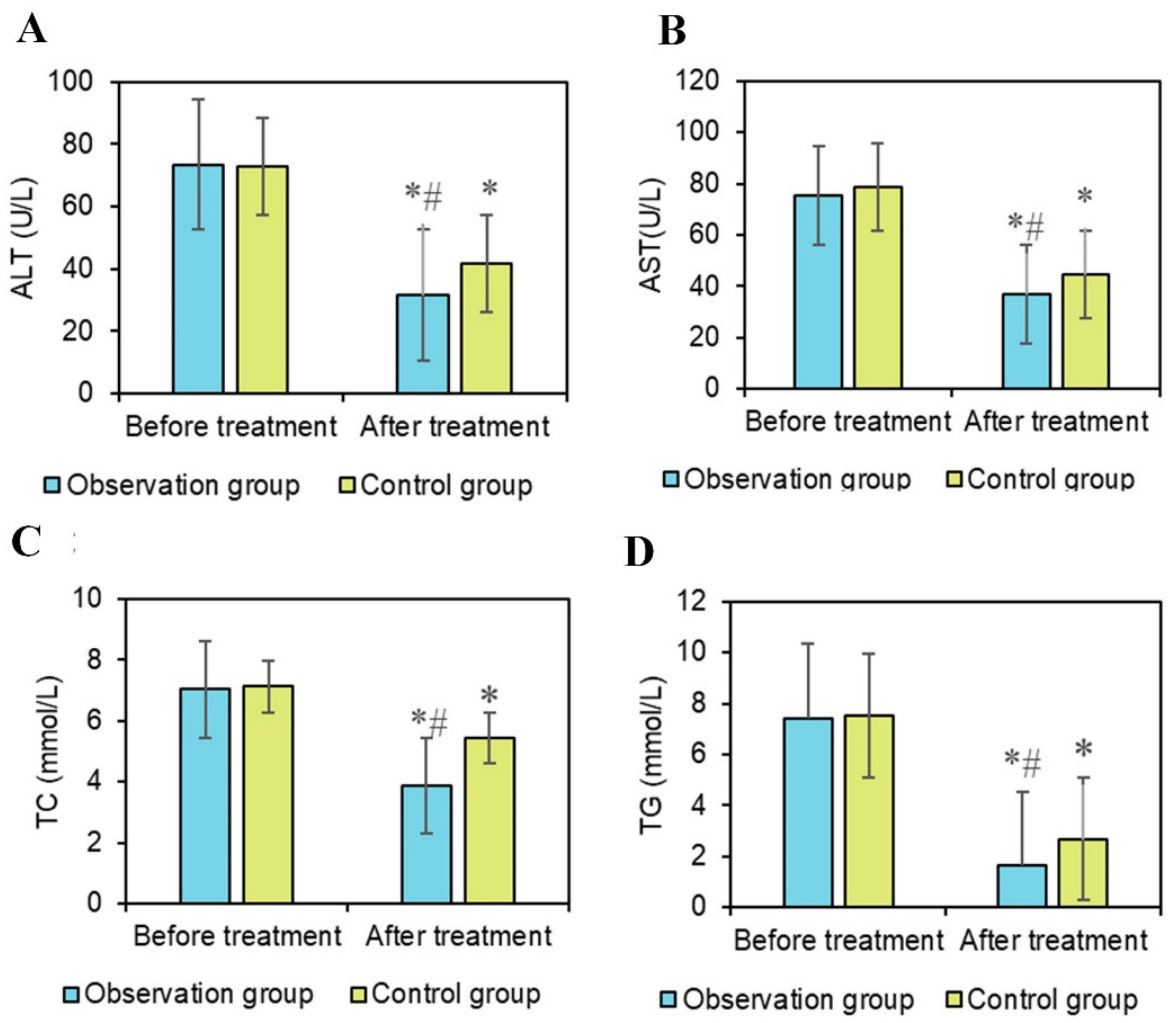
** Comparison of liver function indicators.** Note: A: ALT; B: AST; C: TC; D: TG; #*P*<0.05 vs. before treatment; *P<0.05 vs. OG.

Serum biochemical indicators in liver cancer patients are typically influenced by liver function and metabolism. Serum ALT and AST are enzymes present in liver cells, primarily involved in amino acid metabolism 29. Elevated levels of ALT and AST both reflect liver cell damage. Research by Chen et al. (2022) 30 indicated that AST serves as a prognostic indicator for liver cancer patients. Li et al. (2017) 31 found a significant association between ALT and liver disease. In the results of this study (Figures 6A and 6B), there was little difference in the changes in AST and ALT levels between the pretreatment groups. After treatment, both AST and ALT levels significantly decreased compared to before treatment (*P* < 0.05). Additionally, the AST levels after OG treatment were significantly lower than in the CG (*P* < 0.05). There were neglectable changes in AST and ALT levels between the two groups before treatment. After treatment, the AST levels in both groups markedly decreased, indicating a remarkable therapeutic effect on improving AST levels. The post-treatment AST levels in OG were dramatically inferior to those in CG, suggesting that the combination of conventional chemotherapy and PDT with HPD nanoparticles had a more pronounced effect on reducing AST, potentially reflecting a more effective protection of liver function by this combined therapy. Furthermore, the post-treatment ALT levels in OG were substantially inferior to those in CG, indicating that the combined treatment of conventional chemotherapy with HPD nanoparticles PDT had a more prominent effect on reducing ALT levels.

The changes in blood lipid levels may reflect the overall metabolic status of patients and the impact of treatment on the metabolism of liver cancer patients. Interventions aimed at reducing very low-density lipoprotein secretion have been shown to alleviate lipid abnormalities, as indicated by Heeren and Scheja (2021) 32. Triglycerides (TG) are formed by combining one molecule of glycerol with three molecules of fatty acids. They are one of the main forms of storage in the body and a major source of energy. TG is primarily stored in adipocytes and provides energy to the body when needed. Elevated TG levels may be associated with metabolic diseases such as obesity, hypertension, and diabetes, and are also a risk factor for cardiovascular disease 33. Total cholesterol (TC) is a major component of cell membranes and is crucial for normal cellular structure and function 34. However, high TC levels may increase the risk of cardiovascular disease, particularly when LDL-C levels are elevated. In Figure 6C and 6D, there were neglectable changes in TG and TC levels between groups pre-treatment, but after treatment, they were notably decreased versus before treatment (*P* < 0.05), with the post-treatment levels in OG being markedly inferior to those in CG (*P* < 0.05). These results indicate that notable changes occurred in the lipid levels of patients during treatment. The decreased levels of TG and TC may be related to the effectiveness of the treatment and the improvement in the overall metabolic status of the patients, suggesting that combined treatment with HPD nanomedicine PDT may have better effects in regulating patients' lipid metabolism.

## Conclusion

This study demonstrated that patients in the conventional chemotherapy combined with HPD nanomedicine PDT group exhibited more pronounced tumor shrinkage, necrosis, and alterations in biological characteristics after treatment. Compared with CG, drastic differences were observed in Bcl-2 and Bax protein expression rates, as well as in AST and ALT post-treatment in OG, with a better survival rate observed in OG.

These findings indicate that conventional chemotherapy combined with HPD nanomedicine PDT based on gadopentetate dimeglumine-enhanced MRI has remarkable therapeutic efficacy in patients with liver cancer. PDH nanomedicine therapy for liver cancer can effectively promote cancer cell apoptosis, improve liver function indicators, and enhance the long-term survival rate of patients. This combined treatment strategy not only improves therapeutic efficacy but also reduces adverse reactions to conventional treatment, providing new insights for future research in liver cancer treatment.

However, the study also has several limitations, including a relatively small sample size, single-center study design, and lack of validation in a broader patient population. Future research could further expand the sample size, adopt a multicenter design to validate the effectiveness and safety of this combination therapy strategy on a larger scale. Additionally, further investigation into the treatment mechanism and potential molecular biology basis could be considered.

## Figures and Tables

**Table 1:** Efficacy evaluation

Curative effect	Symptom manifestations
Complete remission	Complete disappearance of lesions after treatment, maintained for at least 4 weeks;
Partial remission	Reduction of lesions by 50% or more, maintained for at least 4 weeks;
Improvement	Reduction of lesions by 25% to 49%, with no new lesions observed;
Stable disease	Reduction of tumor lesions by less than 25%, with no new lesions observed;
Disease progression	Increase of lesions by 25% or more, or appearance of new lesions.

## References

[1] Sucularli C (2022). Identification of BRIP1, NSMCE2, ANAPC7, RAD18 and TTL from chromosome segregation gene set associated with hepatocellular carcinoma. Cancer Genet.

[2] Qian H, Chao X, Williams J (2021). Autophagy in liver diseases: a review. molecular aspects of medicine.

[3] Cheng K, Cai N, Zhu J (2022). Tumor-associated macrophages in liver cancer: from mechanisms to therapy. Cancer Communications (London, England).

[4] Li X, Ramadori P, Pfister D (2021). The immunological and metabolic landscape in primary and metastatic liver cancer. Nature Reviews Cancer.

[5] Hengrui L (2023). An example of toxic medicine used in Traditional Chinese Medicine for cancer treatment. J Tradit Chin Med.

[6] Shim PJ, Zeitouni NC (2020). Photodynamic therapy for extramammary paget's disease: a systematic review of the literature. Photodiagnosis and Photodynamic Therapy.

[7] Gunaydin G, Gedik ME, Ayan S (2021). Photodynamic therapy-current limitations and novel approaches. Frontiers in Chemistry.

[8] Liu H (2020). Effect of Traditional Medicine on Clinical Cancer. Biomedical Journal of Scientific & Technical Research.

[9] Cheng X, Wei Y, Jiang X (2022). Insight into the prospects for tumor therapy based on photodynamic immunotherapy. Pharmaceuticals (Basel, Switzerland).

[10] Wahnou H, Youlyouz-Marfak I, Liagre B (2023). Shining a light on prostate cancer: photodynamic therapy and combination approaches. Pharmaceutics.

[11] Sioud M, Zhang Q (2023). Precision killing of M2 macrophages with phage-displayed peptide-photosensitizer conjugates. Cancers.

[12] Fowler AM, Strigel RM (2022). Clinical advances in PET-MRI for breast cancer. The Lancet. Oncology.

[13] Rivera D (2022). Emerging role for 7T MRI and metabolic imaging for pancreatic and liver cancer. Metabolites.

[14] Rompianesi G, Pegoraro F, Ceresa CD (2022). Artificial intelligence in the diagnosis and management of colorectal cancer liver metastases. World Journal of Gastroenterology.

[15] Park S, Cho E, Chueng SD (2023). Aptameric fluorescent biosensors for liver cancer diagnosis. Biosensors.

[16] Witt JS, Rosenberg SA, Bassetti MF (2020). MRI-guided adaptive radiotherapy for liver tumours: visualising the future. The Lancet Oncology.

[17] Areseney AL, Kanaey SV, Barchk AS (2007). Use of endotracheobronchial surgery in conjunction with radiochemotherapy for advanced non-small lung cancer. Voprosy Onkologii.

[18] Cuneo KC, Herr DJ (2023). Advances in radiation therapy for primary liver cancer. Surgical Oncology Clinics of North America.

[19] Yang T, Luo Y, Liu J (2023). A novel signature incorporating lipid metabolism- and immune-related genes to predict the prognosis and immune landscape in hepatocellular carcinoma. Frontiers in Oncology.

[20] Heidary Pour A, Mansouri N, Kolahi AA (2022). Diagnostic of cytokeratin-19 gene expression in iranian breast cancer patients. Cellular and Molecular Biology (Noisy-le-Grand, France).

[21] Yu G, Rice S, Heer R (2023). Photodynamic Diagnosis-guided Transurethral Resection of Bladder Tumour in Participants with a First Suspected Diagnosis of Intermediate- or High-risk Non-muscle-invasive Bladder Cancer: Cost-effectiveness Analysis Alongside a Randomised Controlled Trial. Eur Urol Open Sci.

[22] Heer R, Lewis R, Duncan A (2022). Photodynamic versus white-light-guided resection of first-diagnosis non-muscle-invasive bladder cancer: PHOTO RCT. Health Technol Assess.

[23] Chong ML, Knight J, Peng G (2023). Integrated exome sequencing and microarray analyses detected genetic defects and underlying pathways of hepatocellular carcinoma. Cancer Genet.

[24] Albillos A, de Gottardi A, Rescigno M (2020). The gut-liver axis in liver disease: pathophysiological basis for therapy. Journal of Hepatology.

[25] Kong WD, Cao JM, Xu J (2012). Impact of low versus conventional doses of chemotherapy during transcatheter arterial chemo-embolization on serum fibrosis indicators and survival of liver cancer patients. Asian Pac J Cancer Prev.

[26] Liu C, Xie Y, Li X (2021). Folic acid/peptides modified PLGA-PEI-PEG polymeric vectors as efficient gene delivery vehicles: synthesis, characterization and their biological performance. Molecular Biotechnology.

[27] Moffatt S, Cristiano RJ (2006). Uptake characteristics of NGR-coupled stealth PEI/pDNA nanoparticles loaded with PLGA-PEG-PLGA tri-block copolymer for targeted delivery to human monocyte-derived dendritic cells. International Journal of Pharmaceutics.

[28] Liang B, He ML, Xiao ZP (2008). Synthesis and characterization of folate-PEG-grafted-hyperbranched-PEI for tumor-targeted gene delivery. Biochemical and Biophysical Research Communications.

[29] Feng H, Chu D, Li Z (2018). A DOX-loaded polymer micelle for effectively inhibiting cancer cells. RSC Advances.

[30] Chen Q, Li M, Chen J (2022). AST·MLR index and operation injury condition are novel prognostic predictor for the prediction of survival in patients with colorectal cancer liver metastases undergoing surgical resection. BMC Cancer.

[31] Li TC, Li CI, Liu CS (2017). Interaction and joint effect of ALT and chronic liver disease on liver cancer in type 2 diabetes patients. Oncotarget.

[32] Heeren J, Scheja L (2021). Metabolic-associated fatty liver disease and lipoprotein metabolism. Molecular Metabolism.

[33] Taher J, Baker C, Alvares D (2018). GLP-2 dysregulates hepatic lipoprotein metabolism, inducing fatty liver and VLDL overproduction in male hamsters and mice. Endocrinology.

[34] Parlevliet ET, Wang Y, Geerling JJ (2012). GLP-1 receptor activation inhibits VLDL production and reverses hepatic steatosis by decreasing hepatic lipogenesis in high-fat-fed APOE*3-Leiden mice. PloS One.

